# *Ribes nigrum* L. Extract-Mediated Green Synthesis and Antibacterial Action Mechanisms of Silver Nanoparticles

**DOI:** 10.3390/antibiotics11101415

**Published:** 2022-10-14

**Authors:** Zaruhi Hovhannisyan, Marina Timotina, Jemma Manoyan, Lilit Gabrielyan, Margarit Petrosyan, Barbara Kusznierewicz, Agnieszka Bartoszek, Claus Jacob, Mikayel Ginovyan, Karen Trchounian, Naira Sahakyan, Muhammad Jawad Nasim

**Affiliations:** 1 Division of Bioorganic Chemistry, School of Pharmacy, Saarland University, 66123 Saarbruecken, Germany; 2 Department of Medical Biochemistry and Biotechnology, Russian-Armenian University, 0051 Yerevan, Armenia; 3 Department of Biochemistry, Microbiology and Biotechnology, Biology Faculty, Yerevan State University, 0025 Yerevan, Armenia; 4 Department of Food Chemistry, Technology and Biotechnology, Faculty of Chemistry, Gdańsk University of Technology, 80-233 Gdańsk, Poland; 5 Research Institute of Biology, Yerevan State University, 0025 Yerevan, Armenia

**Keywords:** silver nanoparticles, *Ribes nigrum*, natural products, phytochemical investigation, antimicrobial

## Abstract

Silver nanoparticles (Ag NPs) represent one of the most widely employed metal-based engineered nanomaterials with a broad range of applications in different areas of science. Plant extracts (PEs) serve as green reducing and coating agents and can be exploited for the generation of Ag NPs. In this study, the phytochemical composition of ethanolic extract of black currant (*Ribes nigrum*) leaves was determined. The main components of extract include quercetin rutinoside, quercetin hexoside, quercetin glucuronide, quercetin malonylglucoside and quercitrin. The extract was subsequently employed for the green synthesis of Ag NPs. Consequently, *R. nigrum* leaf extract and Ag NPs were evaluated for potential antibacterial activities against Gram-negative bacteria (*Escherichia coli* ATCC 25922 and kanamycin-resistant *E. coli* pARG-25 strains). Intriguingly, the plant extract did not show any antibacterial effect, whilst Ag NPs demonstrated significant activity against tested bacteria. Biogenic Ag NPs affect the ATPase activity and energy-dependent H^+^-fluxes in both strains of *E. coli*, even in the presence of *N,N’*-dicyclohexylcarbodiimide (DCCD). Thus, the antibacterial activity of the investigated Ag NPs can be explained by their impact on the membrane-associated properties of bacteria.

## 1. Introduction

The emergence of antibiotic resistance is a serious challenge for both human and veterinary medicine. Several mechanisms of antibiotic-resistance development are known [1,2,3]. Although there are some approaches to overcome this threat, no final solution is found yet. The antibacterial properties of silver date back to ancient times [4]. Silver (positively charged silver ions (Ag^+^), when dissolved in aqueous environment) provides strong antimicrobial activity against a wide spectrum of microorganisms. Silver ions serve as multifunctional agents which, for instance, can produce pores in bacterial cell walls through interaction with the peptidoglycan components [5]. The other mechanism of antimicrobial action of silver cations involves its ability to penetrate the bacterial cell membrane, which leads to the inhibition of cellular respiration and, consequently, the generation of the reactive oxygen species (ROS). Moreover, Ag^+^ is highly toxic to microorganisms due to its ability to disrupt not only DNA but also their replication cycle [5].

There are different approaches for generating Ag nanoparticles (Ag NPs) such as physical (grinding), chemical and biological methods. All these methods have their specific advantages and disadvantages. Although the physical methods produce very stable and small Ag NPs in high concentrations, this process is associated with several disadvantages, such as consumption of energy, wear and tear of grinding balls and bowl, contamination of the nanoparticles, etc. The chemical methods of Ag NPs generation are highly harmful to the environment. Biological methods provide several advantages, such as low cost and ease of implementation. The process is considered green, environmentally and eco-friendly [6,7,8,9]. Natural products, especially those of plant origin, provide reducing as well as coating characteristics which can be utilized for the generation and stabilization of metal nanoparticles [7,9,10,11,12,13].

Recent years have witnessed a considerable interest in the application of green Ag NPs in food packaging, textiles, cosmetics and the biomedical-related product industry (wound dressing components, implants) [7,14,15,16]. Ag NPs provide antimicrobial, anticancer, antioxidant, anti-inflammatory, wound healing and antimalarial activities [17]. The other major applications of silver nanoparticles include diagnostic (as biological tags in biosensors, assays, and quantitative detection), conductive (in conductive inks, pastes, and fillers), optical (metal-enhanced fluorescence and surface-enhanced Raman scattering), and household (pesticides and wastewater treatment) [17]. Ag NP produced from the leaf extract of *Vitex negundo* was reported to arrest HCT-15 cells at G_0_/G_1_ and G_2_/M phases and thereby serve as a potential antitumor agent against human colon cancer cell line HCT15. Another antiproliferative mechanism of Ag NPs against colon cancer cell lines involves the reduction of DNA synthesis by arresting G_0_/G_1_ phase, ultimately triggering apoptosis [18,19]. In addition, the secondary metabolites present in natural products not only provide biological activities but also reduce the side effects of the synthesized Ag NPs and make them more amenable for medical applications [7,20]. Furthermore, several natural products, such as water-soluble polyphenolic substances, serve as chelating agents which adsorb easily at the surface of NPs, enhance their stability and protect them from aggregation [7,21].

*Ribes nigrum* L. (black currant) is a deciduous shrub, which belongs to the family Grossulariaceae and is extensively consumed throughout the world for the dietary management of various diseases [22,23]. The efficacy of this particular natural product in the management of various diseases is associated with the presence of a broad spectrum of biologically active components, including strong antioxidants and reducing agents. The leaves of *R. nigrum* are frequently employed in European traditional medicine for the prophylaxis and treatment of various deviations in human metabolism [22,23]. Black currant leaf infusions have been utilized for the efficient removal of toxins from the body and the regulation of kidney function. Its extracts are used as diaphoretic and diuretic agents and for treating inflammatory disorders, such as rheumatic disease [24]. Proanthocyanidins isolated from leaves of *R. nigrum* are reported to exhibit excellent in vivo anti-inflammatory activity in rat models [25]. The phytochemical analysis of blackcurrant fruit methanolic extracts by HPLC has revealed the presence of antioxidant components, such as epigallocatechin (EGC) and epigallocatechin–3–gallate (EGCG) [26]. The phytochemical composition of fruits of *R. nigrum* has been extensively studied and described in the literature [27]. Intriguingly, the content of active phenolic compounds, which are mainly responsible for antioxidant and other biological activities of this plant are much higher in leaf extracts than in fruits [24]. Staszowska-Karkut and Materska [24] have recently reported the presence of 19 phenolic compounds in the leaves of *R. nigrum*. The aqueous–alcoholic leaf extract of *R. nigrum* is reported to provide antimicrobial activity, which is lower than fruit extract [27]. There is no literature concerning the acute toxicity, genotoxicity, reproductive and developmental toxicity or carcinogenicity of this plant extract [28]. Literature reveals several reports on the green synthesis of Ag NPs using *R. nigrum* fruit or pomace extracts [29,30]; however, there is no literature about the utilization of leaf extracts. 

The current study is mainly focused on the understanding of the underlying mechanisms of antibacterial activities of Ag NPs, produced by the leaf extracts of *R. nigrum* harvested from high-altitude Armenian landscape against *Escherichia coli* strains (including drug-resistant ones).

## 2. Results

### 2.1. The Total Phenolic-Flavonoid Composition of R. nigrum Extracts

The total flavonoid and phenol contents in *R. nigrum* extracts were estimated from the calibration curve of quercetin and gallic acid, respectively. The total flavonoid and phenolic contents of 35.42 ± 1.52 mg quercetin equivalent (QE)/g DW) and 84.1 ± 1.6 mg gallic acid equivalent (GAE)/g DW) were quantified, respectively. The presence of high flavonoid and phenolic content in the *R. nigrum* extract indicates its potential as a reducing agent. 

### 2.2. Identification of Major Polyphenols in R. nigrum Extract

**Figure 1:**
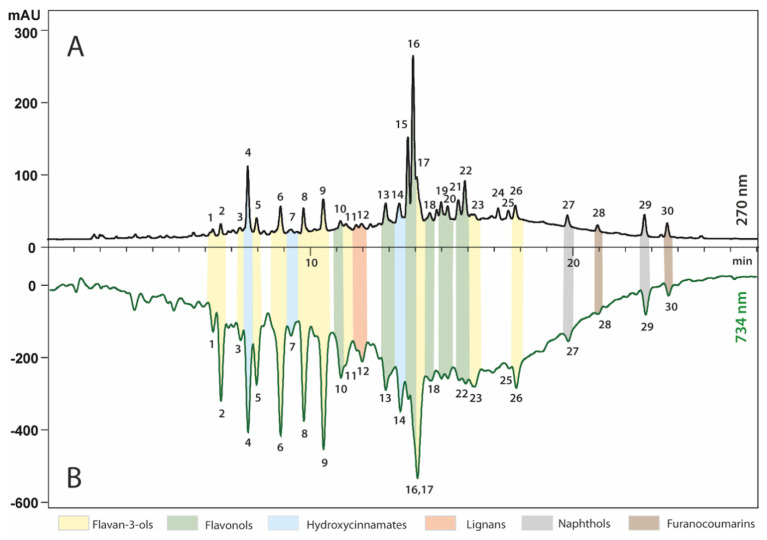
HPLC profiles of extracts from *R. nigrum* recorded before (270 nm, Panel **A**) and after (734 nm, Panel **B**) post-column derivatization with ABTS. For identity of peaks, see Table 1.

The HPLC results have revealed the presence of about 30 major constituents comprising mainly of flavan-3-ols, flavonols, hydroxycinnamates, lignans, naphthols and furanocoumarins (Figure 1). Each compound was assigned to one of the above-mentioned groups by the careful analysis of its UV–Vis spectrum. Finally, the compounds were identified based on the full scan MS, MS2 spectra, obtained for major *m/z* signals recorded in negative ion mode, retention time, and bibliography (Table 1).

Compounds **4**, **7**, **14**, **24** and **25** were classified as hydroxycinnamic acids derivatives. Compounds **4** and **7** provided pseudo-molecular ions at *m/z* 353.08772 (C_16_H_17_O_9_). The further MS2 fragmentation spectra revealed the identity of these two compounds as caffeoyl quinic acid isomers. In the case of compounds **14**, **24** and **25**, it was not possible to assign any particular structure but the presence of ion at *m/z* 163.03952 (C_9_H_7_O_3_) in MS2 spectra hinted at the presence of coumaric acid derivatives.

Compounds **10**, **13**, **15**, **16** and **18–22** were classified as flavonols. Compound **10** provided the base peak ion in MS spectrum at *m/z* 477.10395 (C_22_H_21_O_12_) followed by the cleavage of hexose moiety (−162 amu) in MS2 spectrum. Thus, compound **10** may tentatively be assigned as rhamnetin glucoside. Compounds **13**, **15**, **16** and **18–22** yielded the same fragmentation ion at *m/z* 301.0348 (C_15_H_9_O_7_), suggesting the presence of quercetin derivatives. Compounds **18**, **20** and **21** generated the same pseudo-molecular ion [M−H]^−^ at *m/z* 433.07765 (C_20_H_17_O_11_) which in MS2 were shown to lose 132 amu corresponding to pentose moiety. Thus, these compounds were assigned as quercetin pentoside isomers. In the case of other quercetin derivatives, according to the MS and MS2 spectra the loss of rhamnose-glucose (−308 amu), hexose (−162 amu), glucuronide (−176 amu), malonyl hexose (−248 amu) and rhamnose (−146 amu) moieties were observed for compounds **13**, **15**, **16**, **19** and **22**, respectively. Thus, these compounds were tentatively identified as quercetin rutinoside, quercetin hexoside, quercetin glucuronide, quercetin malonylglucoside and quercitrin, respectively.

Compounds **1–3**, **5**, **6**, **8**, **9**, **11**, **17**, **23** and **26** were classified as flavan-3-ols. Intriguingly, the peaks of compounds **5** and **8**, with almost similar precursor ion [M–H]^−^ of *m/z* 289.07171 and 289.07163, respectively, were detected at different RT values of 7.54 and 9.30 min. Having the same molecular formula C_15_H_14_O_6_ and fragmentation pattern, these compounds were proposed to be (+)-catechin and (−)-epicatechin. Peak of compound **2** was obtained [M–H]^−^ ion at *m/z* 305.06659 (C_15_H_13_O_7_) and has shown to produce fragment ions at *m/z* 167.0322 and 125.0248 by retro-Diels-Alder fragmentation, therefore, compounds like (+)-gallocatechin or (-)-epigallocatechin were expected. Likewise, an epicatechingallate (**17**, C_22_H_18_O_10_) has also been tentatively identified due to the presence of the deprotonated ion at *m/z* 441.08274 and the fragment ions which were observed after the loss of a galloyl residue at *m/z* 289.07121 [(−)-epicatechin–H]^−^ or as the deprotonated gallic acid at *m/z* 169.01491. Compounds **1**, **3** and **6** with *m/z* values of around 577.13 (C_30_H_25_O_12_) were identified as dimers of (*epi*)catechin. Peaks of compounds **23** and **26** with *m/z* values of 729.14679 (C_37_H_29_O_16_) and 881.15809 (C_44_H_33_O_20_), with similar MS2 patterns were tentatively identified as galloylated and digalloylated dimeric B-type proanthocyanidins, respectively. Compounds **9** and **11** with *m/z* values of 863.18375 (C_45_H_35_O_18_) and 1153.26251 (C_60_H_49_O_24_) were tentatively assigned as trimeric and tetrameric A-type pro-anthocyanidins, respectively.

Compound **12** showed the base peak ion in the MS spectrum at *m/z* 521.20294 (C_26_H_33_O_11_) followed by the cleavage of hexose moiety (−162 amu) in MS2 spectrum. Thus, this compound was classified as a lignan and tentatively assigned as lariciresinol glucoside.

Intriguingly, compounds **27** and **29** classified as naphthols with precursor ionic peaks at *m/z* 377.12408 (C_19_H_21_O_8_) and *m/z* 419.13471 (C_21_H_23_O_9_), respectively, yielded the same fragmentation ion at *m/z*215.07082 (C_13_H_11_O_3_), suggesting these compounds as musizin (nepodin) derivatives. The loss of hexose (−162 amu) and acetyl hexose (−204 amu) moieties from these compounds has led us to tentatively assign them as musizin glucoside and musizin acetyl glucoside, respectively.

A similar pattern of fragmentation has also been observed for compounds **28** and **30**. The fragmentation of precursor ions at *m/z* values of 407.13468 (C_20_H_23_O_9_) and 449.14526 (C_22_H_26_O_10_) for compounds **28** and **30**, respectively, have provided the same aglycon ion at *m/z* 245.08138 (C_14_H_13_O_4_) due to the loss of hexose and acetyl-hexose moieties. Therefore, these compounds were tentatively identified as marmesin glucoside and marmesin acetyl glucoside, respectively.

### 2.3. Radical Scavenging Capacity of R. nigrum Extract

The potential antioxidant activity of *R. nigrum* extract was confirmed by DPPH assay. The radical scavenging activity of plant extract was compared with that of catechin (IC_50_ = 12.62 ± 0.8 µg/mL), which is a well-known antioxidant and exhibits excellent DPPH scavenging properties. In our study, *R. nigrum* extract provided an IC_50_ value of 63.59 ± 1.63 µg/mL (R² = 0.9462) which clearly indicated that the extract was able to scavenge 50% of the radicals at the concentration of around 63.59 µg/mL.

### 2.4. Antioxidant Profiling by HPLC Coupled Post-Column Derivatization

Post-column derivatization of analytes with ABTS reagent was performed during HPLC analysis of *R. nigrum* extract. This investigation has allowed us to identify the specific compounds responsible for the antioxidant activity. As observed in the ABTS colorimetric tests, the reduction reaction has led to a significant shift in the UV-Visible spectrum, resulting in ABTS reagent absorption change (discoloration). The presence of antioxidants in the eluate has provided negative peaks in the chromatogram, recorded after derivatization at 734 nm (Figure 1B). The profile obtained after derivatization indicates that almost all of the compounds identified in *R. nigrum* extract exhibit antioxidant activity. The highest antioxidant activity was observed for flavan-3-ols, followed by hydroxycinnamates and flavonols.

### 2.5. Metal Chelating Capability of R. nigrum Leaf Extract

*R. nigrum* leaf extract provided 56.53 ± 0.7% of metal chelating activity, whilst the same concentration of positive control (EDTA) provided 90 ± 0.8%.

### 2.6. Production of Silver Nanoparticles Using Plant Extract

**Figure 2:**
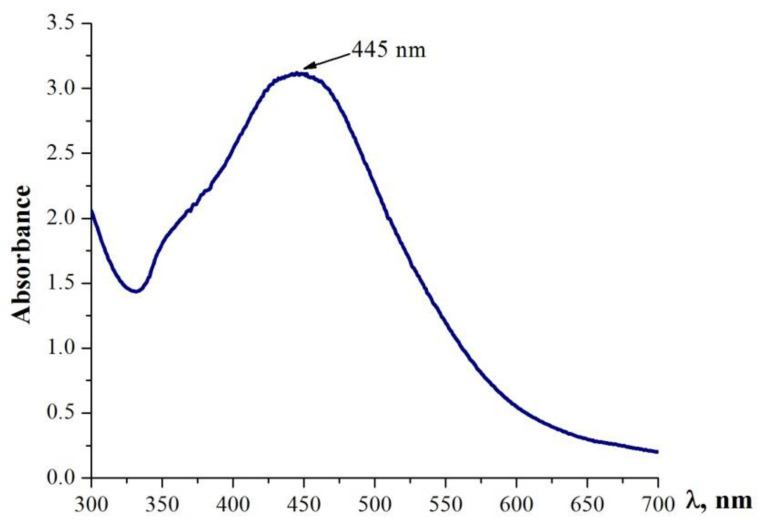
UV-Vis spectrum of reaction mixture containing biosynthesized Ag NPs.

The phytochemical profiling of *R. nigrum* leaf extract affirmed the presence of polyphenols whose reducing nature paved the way for the green synthesis of Ag NPs. Experimental data have shown that the synthesis of nanoparticles occurs in the presence of light. The formation of Ag NPs was confirmed by exploiting UV–Vis spectroscopy (Figure 2). The results have indicated the emergence of an absorbance peak at around 445 nm, which is specific for Ag NPs in the samples (Figure 2) [9].

**Figure 3:**
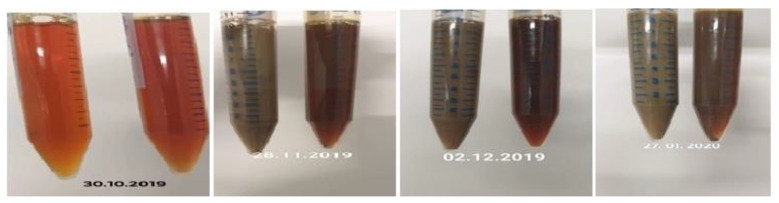
The change in the color of nano-suspensions with the passage of time.

In our examination, the color of the reaction mixture changed from light yellow to brown in the presence of the plant extract after 18 h of incubation (Figure 3). This change in color affirmed the production of Ag NPs, whilst the change of color from brown to dark brown or black could indicate the possible oxidation of Ag NPs as a result of the interaction of Ag NPs with oxygen, leading to the formation of silver oxides as described in the literature [31] and will be investigated in the follow-up project using X-Ray Diffraction (XRD). This change in color from brown to dark brown or black may also be due to possible aggregation [32]. No color change was observed in the absence of plant extract. Since photon energy is necessary for the formation of Ag NPs from Ag^+^ in the presence of plant extracts, the experiments were performed in the presence of light [33].

### 2.7. Characterization of Ag NPs

The biosynthesized Ag NPs were characterized by analytical techniques such as dynamic light scattering (DLS), zeta potential (ZP), scanning electron microscopy (SEM), transmission electron microscopy (TEM) analysis and inductively coupled plasma-optical emission spectrometry (ICP-OES). 

Dynamic light scattering (DLS) measures the average size of nanoparticles based on the method of laser beam diffraction [34]. The results of DLS analysis provide a Z-average size of nanoparticles as 61.14 ± 0.17 d.nm. Polydispersity index (PDI) indicates the heterogeneity of a sample based on size [35]. The value of PDI ranges from 0.0 for a very homogeneous sample to 1.0 for a very heterogeneous sample. In our experiments, this value ranged from 0.1 to 0.4 for tested samples, which mean that the investigated suspension was somewhat polydisperse [30]. This relatively high value of PDI was expected as the sample was only dispersed in aqueous medium and no surfactants were utilized to have a more practical values when applied in biological testing. 

The Z-potential (ZP) values provide information about the nanoparticle surface charge and the probability of their aggregation [36,37] which in our sample was observed to be around −19.2 ± 1.02 mV. The obtained ZP value indicated that the investigated NPs were prone to agglomeration, which may be the reason for further aggregation.

**Figure 4:**
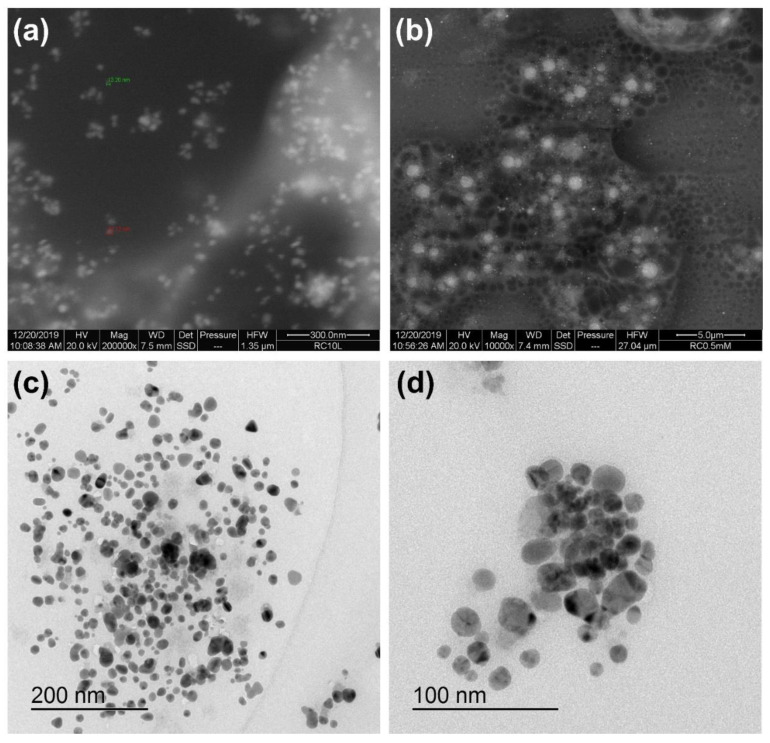
SEM images of Ag NPs (panels **a** and **b**). TEM Images of Ag NPs (panels **c** and **d**). See text for details.

The SEM and TEM were performed to determine the particle size and shapes as described above. SEM images revealed that Ag NPs range in size from 1 to 50 nm (Figure 4a,b). TEM image has also confirmed the above-mentioned sizes of the investigated NPs (Figure 4c,d). Moreover, it affirmed that the particles were spherical in shape with low agglomeration. EDX revealed a spectral signal in silver region affirming the presence of Ag NPs [38]. The spectral signals of carbon, oxygen, chlorine, and sulfur were also observed, which are generally present in biological material (plant extracts of *R. nigrum*) [7].

The total silver content was determined by employing inductively coupled plasma-optical emission spectrometry (ICP-OES) which provided a value of 83.50 ± 3.32 µg/mL [39]. Considering the operating principles of ICP-OES, most likely, only small particles may have been detected, and the large particles may have been discarded by the instrument.

### 2.8. Effect of Biogenic Ag NPs on Bacterial Growth Rate, F_O_F_1_-ATPase Activity, and H^+^-Fluxes through the Membrane in Escherichia coli ATCC 25922 and Drug-Resistant E. coli pARG-25 Strains

Our previous investigations have shown antibacterial activity of biogenic Ag NPs against different Gram-positive and Gram-negative bacteria [7,9]. Intriguingly, the Gram-negative bacteria were more susceptible to this agent, perhaps due to the possible interaction of these particles with bacterial cell walls [7,9]. Therefore, in order to investigate the mechanism of antibacterial activities, two strains of *Escherichia coli* i.e., ATCC 25922 and drug-resistant *E. coli* pARG-25 were employed in this study.

**Figure 5:**
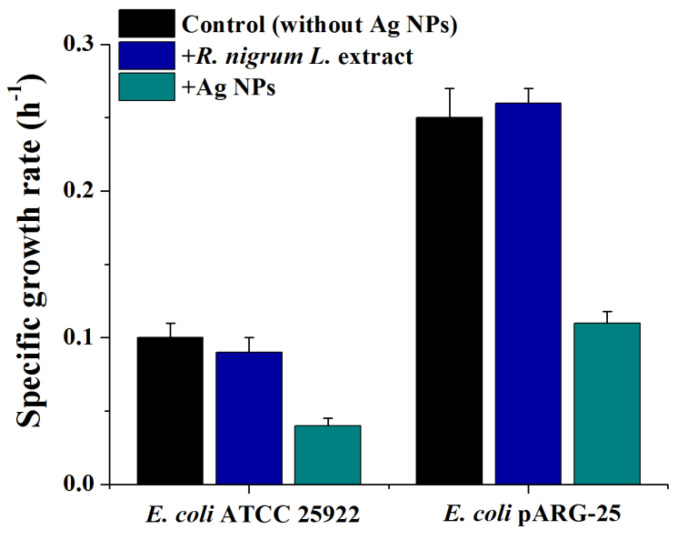
Impact of biogenic Ag NPs on specific growth rate of *E. coli* ATCC 25922 and kanamycin-resistant *E. coli* pARG-25 strains.

Since the disk-diffusion method revealed an MIC value of 10 µg/mL for Ag NPs, a similar concentration was employed to investigate the antibacterial mechanisms of biogenic Ag NPs against *E. coli* ATCC 25922 and kanamycin resistant *E. coli* strains. It should be mentioned that *R. nigrum* extract did not show any antibacterial effect (Figure 5). At a concentration of 10 µg/mL, the biogenic Ag NPs decreased the growth rate of *E. coli* wild type and drug-resistant strain by ~2.5 and 2.3-fold, respectively, in comparison with control cells (without Ag NPs addition) as presented in Figure 5. Thus, the biogenic NPs have demonstrated excellent antibacterial activity against both tested strains of bacteria.

**Figure 6:**
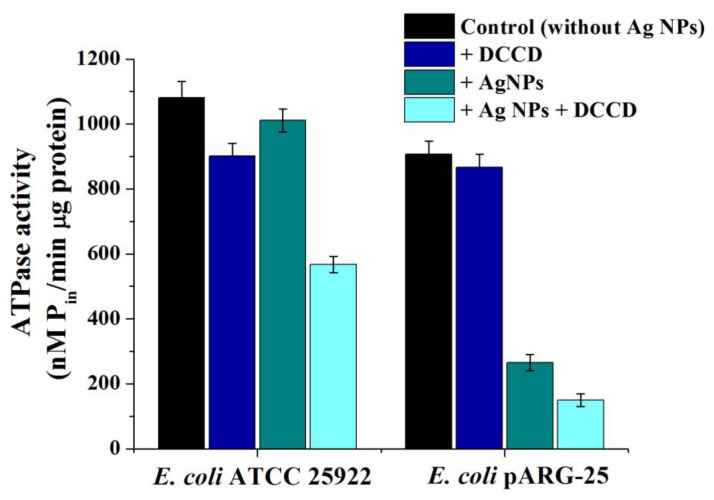
The impact of biogenic Ag NPs on ATPase activity of *E. coli* ATCC 25922 and kanamycin-resistant *E. coli* pARG-25 membrane vesicles.

To understand the mechanisms underlying the antibacterial activity of the biogenic Ag NPs, H^+^-translocating F_O_F_1_-ATPase activity and H^+^-fluxes through the bacterial membrane were determined. In the case of *E. coli* ATCC 25922, no significant effect of Ag NPs on the total F_O_F_1_-ATPase activity in membrane vesicles was observed, whilst the addition of the Ag NPs decreased ATPase activity in drug-resistant strain by ~3.4 fold, in comparison with control (without Ag NPs addition), as shown in Figure 6. Moreover, DCCD-sensitive ATPase activity in *E. coli* pARG-25 decreased in the presence of Ag NPs by ~6 fold (Figure 6). In the case of *E. coli* ATCC 25922, DCCD-sensitive ATPase activity was observed to be ~1.6-fold lower than control cells (Figure 6).

Furthermore, biogenic Ag NPs were observed to affect the H^+^-translocating ATPase which is closely related to the decrease of ATPase activity as observed in the presence and absence of DCCD, an inhibitor of H^+^-translocation systems. Moreover, the F_O_F_1_-ATPase can be considered a target of Ag NPs responsible for the antimicrobial effect of biogenic Ag NPs.

**Figure 7:**
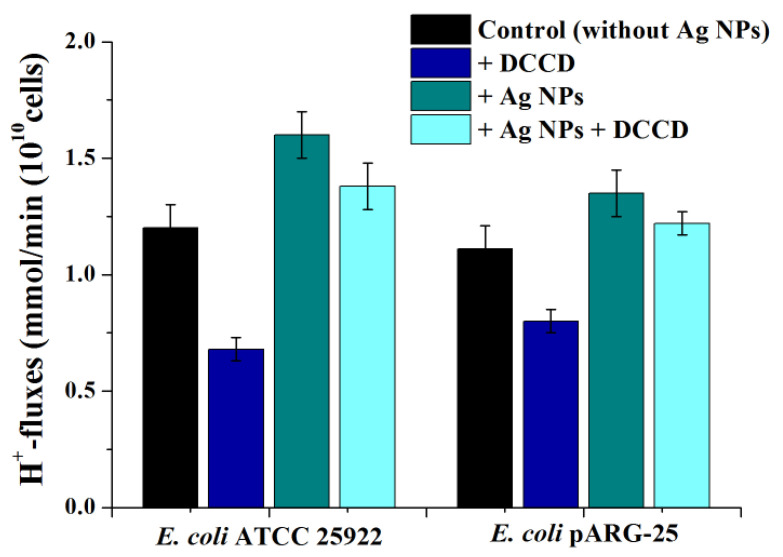
The effect of biogenic Ag NPs on H^+^-fluxes through the *E. coli* ATCC 25922 and *E. coli* pARG-25 membranes.

The analysis of energy-dependent H^+^-fluxes through the bacterial membrane demonstrated that Ag NPs enhanced H^+^-fluxes in both strains of *E. coli* by~1.2–1.3 fold (Figure 7). A decrease in H^+^-fluxes was observed in the presence of DCCD. Moreover, Ag NPs increased DCCD-sensitive H^+^-fluxes by ~2 and 1.5-fold in *E. coli* ATCC 25922 and *E. coli* pARG-25, respectively (Figure 7).

## 3. Discussion

The leaves of *R. nigrum* are widely used in European traditional medicine due to the presence of high content of biologically active phytochemicals including flavonoids and phenolic compounds. Such active moieties are mostly associated with redox character responsible for antioxidant activity. The high content of such active metabolites in *R. nigrum* extract makes them suitable to serve as reducing agent not only in chemical-based tests, but also in the biogenesis of silver nanoparticles. Several other scientific publications affirm the notion that such extract could be employed for the synthesis of Ag NPs [7,10,11,12,15,28,40].

Phenolic fraction of *R. nigrum* extract was investigated for the identification of compounds and the results have revealed the presence of around 30 major constituents comprising mainly of flavan-3-ols, flavonols, hydroxycinnamates, lignans, naphthols and furanocoumarins. The literature data also suggest quercetin derivatives as the main components of *R. nigrum* leaf extracts, which constitute around 80% of polyphenolic compounds on a dry mass basis [41]. The presence of such a variety of active ingredients explains the excellent antioxidant as well as reducing capacity of *R. nigrum* extract. Numerous studies have shown that extracts rich in phenolic content exhibit strong antioxidant activity [7,42,43,44]. Moreover, the post-column derivatization of analytes with ABTS reagent indicated, that almost all of the compounds identified in *R. nigrum* extract exhibit antioxidant activity. 

**Figure 8:**
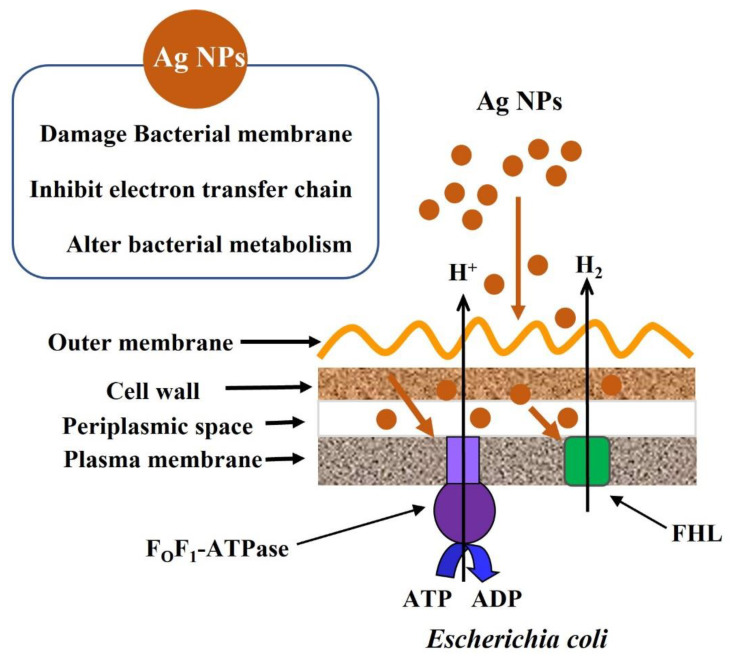
Schematic presentation of possible action mechanisms of Ag NPs on *E. coli*.

Again, due to the high flavonoid and phenolic content, the *R. nigrum* leaf extract expresses the metal chelating ability which is important to stabilize the Ag NPs by preventing their aggregation and agglomeration [45,46]. In our investigations, this capacity was not strong enough to provide the long-term stability of the nanoparticles and eventually, some aggregation was observed which also led to a decrease in the activity of biogenic nanoparticles. This tendency for agglomeration was also confirmed by the obtained ZP values of the investigated samples. The particles have provided excellent antibacterial activities against both strains of *E. coli* perhaps due to the relatively small size and round shapes of nanoparticles and by changing the H^+^-translocating ATPase activity and energy-dependent H^+^-fluxes (Figure 8).

F_O_F_1_-ATPase plays a crucial role in bacterial energetics as it mediates several energy-dependent processes, including ion transport, and regulation of the enzymatic activity of the membrane. F_O_F_1_-ATPase is directly involved in secondary solute transport systems, such as K^+^ uptake, Trk-like or KtrI system forming H^+^/K^+^-exchanging pump. The H^+^/K^+^-exchanging pump, therefore, plays a crucial role to provide antibacterial activity [47]. 

Assuming all of the above-mentioned findings, it is possible to conclude, that the biogenic Ag NPs provide antibacterial activity via the changes in membrane permeability as affirmed by another study using chemically synthesized Ag NPs [48].

## 4. Materials and Methods

### 4.1. Chemicals and Reagents

Folin–Ciocalteu (FC) reagent, ethanol, gallic acid, 2,2-Diphenyl-1-picrylhydrazyl (DPPH), EDTA, AgNO_3_, kanamycin, ampicillin, Mueller–Hinton agar and catechin were purchased from Sigma-Aldrich GmbH (Taufkirchen, Germany). Colloidal Ag, “Silverton” was purchased from “Tonus-Les” Lab (Armenia).

### 4.2. Plant Material Collection, Identification and Extraction

The plant material (*R. nigrum* L.) was collected from Lori province (Armenia, 1600–1650 m a.s.l.) during the fruiting period (July 2019). The identification of the plant was carried out at the Department of Botany and Mycology, Yerevan State University (YSU), Armenia. Plant samples were deposited at the Herbarium of the Department of Botany and Mycology, YSU, Armenia. The collected leaves were washed, dried in the shadow at room temperature and subsequently crushed to obtain the powder, which was stored in a dry and dark place until use. Plant material was extracted using ethanol, as described in the European Pharmacopeia [49]. The obtained dried extracts were stored at 4 °C until further use.

### 4.3. Determination of Total Phenolic and Flavonoid Content

The total phenolic content of plant extracts was measured exploiting the Folin–Ciocalteu (FC) reagent employing a calibration curve of gallic acid (GA) (0–250 µg/mL) using a UV-Vis spectrophotometer (Genesys 10S, Thermo Scientific, Waltham, MA, USA) [13,40,50]. 

The total flavonoid content of *R. nigrum* extract was determined employing AlCl_3_ colorimetric assay utilizing a UV-Vis spectrophotometer (Genesys 10S, Thermo Scientific, Waltham, MA, USA) [42,51]. 

### 4.4. LC-Q-Orbitrap HRMS Analysis

The phytochemical analysis of *R. nigrum* extract was performed using a Dionex Ultimate 3000 UHPLC system (Thermo Scientific ^TM^, Dionex, San Jose, CA, USA) equipped with Synergi ^TM^ Hydro-RP A (150 × 4.5 mm, 4 µm, Phenomenex) column, held at a temperature of 30 °C as described in the literature [52].

Raw data from high-resolution mass spectrometry was elaborated with Compound Discoverer (v. 2.1, Thermo, Waltham, MA, USA), which facilitated the peak recognition, retention times arrangement, profile assignment, and isotope pattern. Major metabolite identification was based on accurate mass and mass fragmentation pattern spectra against MS-MS spectra of compounds available on a customized database of different classes of phytochemicals created on the basis of literature data and implemented in the software. Raw data from three experimental replicates and a blank sample were processed using a workflow presented by Kusznierewicz, Mróz, Koss-Mikołajczyk, and Namieśnik [52].

### 4.5. Post-Column Derivatization with ABTS

Profiles of polyphenols and antioxidants for *R. nigrum* extract were obtained employing the HPLC-DAD system (Agilent Technologies, Wilmington, DE, USA) connected with a Pinnacle PCX Derivatization Instrument (Pickering Laboratories Inc., Mountain View, CA, USA) and UV–Vis detector (Agilent Technologies, Wilmington, DE, USA). The conditions of chromatographic separation were the same as in the case of LC-HRMS analysis.

The chromatograms before derivatization were recorded at 270 nm in DAD detector. The eluate stream from the DAD detector was directed to the post-column derivatization instrument. The post-column derivatization with ABTS reagent was carried out according to methods described in the literature with slight modification [53,54]. A stream of methanolic ABTS solution (1 mM) was introduced to the stream of eluate at a rate of 0.1 mL/min and then directed to the reaction loop (1 mL, 130°C). The antioxidant profiles were recorded in a UV-Vis detector at 734 nm.

### 4.6. 2,2-Diphenyl-1-picrylhydrazyl Free Radical Scavenging Assay

Free radical scavenging assay was performed as described by Hambardzumyan et al. [7]. Catechin was used as a standard.

### 4.7. Chelating Capability of R. nigrum Leaf Extract

Fe^2+^ chelating capability (CC) of the *R. nigrum* leaf extract was determined according to methods described in the literature [15]. EDTA (1 mg/mL) was used, as a reference chelating agent. 

### 4.8. Synthesis of Ag NPs Using R. nigrum Extracts

A stock solution of *R. nigrum* extract was prepared by dissolving 5 mg of plant extract in 10 mL of Milli-Q water (18.2 MΩ·cm at 25 °C) and Ag NPs were synthesized by mixing the solutions of AgNO_3_ (10 mM) and plant extract in 1:9 ratio to achieve a final concentration of 1 mM for AgNO_3_ [6,55]. A control sample excluding plant extract was also prepared similarly. The samples were agitated on a shaker (150 rpm) under dark and light (normal room light) conditions at a temperature of 23 ± 2 °C for 18 h. 

### 4.9. Characterization of Biosynthesized Ag NPs 

The optical properties of Ag NPs were characterized by exploiting a UV-Vis spectrophotometer (Lambda 35, Perkin Elmer, Waltham, MA, USA) [7]. The physical stability of the investigated samples was evaluated by the zeta potential (ξ-potential) measurement and dynamic light scattering (DLS), employing a Zetasizer Nano ZS (Malvern Instruments, Malvern, UK). The size of biosynthesized Ag NPs was determined by Scanning Electron Microscopy (SEM-ZEISS-SUPRA 40/gemini column) equipped with Electron Backscatter Diffraction (EBSD) detector and Energy Dispersive X-ray (EDX) detector -SEM-EDX analysis. Moreover, the size and shape of Ag NPs were evaluated by transmission electron microscopy (LEOL JEM-1400 TEM). The total Ag content was determined by inductively coupled plasma-optical emission spectroscopy (ICP-OES, Horiba JobinYvonUltima 2).

### 4.10. Antibacterial Activity of Biosynthesized Ag NPs

The antibacterial activity of biosynthesized Ag NPs was evaluated against *E. coli* ATCC 25922, and kanamycin-resistant *E. coli* pARG-25 (Scientific-Production Center “ArmBiotechnology”, NAS, Yerevan, Armenia) strains by a disk-diffusion method employing disks with 6 mm in diameter, as described by Hambardzumyan et al., 2020 [7,48]. The Mueller–Hinton agar was exploited for the growth of bacteria. The antibacterial activity of Ag NPs was recorded in terms of Minimum Inhibitory Concentration (MIC). MIC values were recorded after 18 h of incubation at 37 °C. Kanamycin and ampicillin (50 µg/mL) were used as positive controls. Results were compared to the antibacterial activity of colloidal Ag, commercially available under the “Silverton” trademark (“Tonus-Les” Lab, Yerevan, Armenia), and produced by an electrochemical process [48,56].

### 4.11. Growth Kinetics of E. coli ATCC 25922 and E. coli pAPG-25 Strains under the Influence of Biosynthesized Ag NPs

The growth kinetics of *E. coli* ATCC 25922 and *E. coli* pARG-25 strains were monitored in the presence of *R. nigrum* leaf extract. Fresh bacterial strains were isolated from Mueller–Hinton agar plates and transferred to LB broth (pH 7.5) followed by incubation for 18 h at 37 °C. The antibacterial effect of the extract was monitored at the same concentration employed to produce Ag NPs i.e., 0.5 mg/mL. Bacterial growth curves were determined by measuring the turbidity of samples containing bacteria at 565 ± 15 nm every 60 min, exploiting a densitometer (DEN-1B McFarland, Biosan, Latvia) [57]. 

The specific growth rate (μ) of bacteria was calculated according to μ = (lnOD_t_ − lnOD_0_)/t, where OD_0_ represents the initial value of optical density (OD); OD_t_ represents the value of OD after 6 h; μ is expressed as h^−1^ [48].

### 4.12. Determination of H^+^-fluxes 

The H^+^-fluxes through the membrane were determined in whole bacterial cells by employing an appropriate selective electrode (HJ1131B, HANNA Instruments, Portugal. Bacterial cells were transferred into the assay medium containing 150 mmol/L Tris-phosphate buffer (pH 7.5), 0.4 mmol/L MgSO_4_, 1 mmol/L KCl and 1 mmol/L NaCl followed by the addition of 11 mmol/L of glucose. The H^+^-fluxes were expressed in mmol H^+^ per min per 10^10^ cells [48,58]. *N*, *N*’-dicyclohexylcarbodiimide (DCCD) served as an inhibitor of the F_O_F_1_-ATPase and the bacterial cultures were incubated with 0.2 mmol/L of DCCD for 10 min [58].

In order to understand the possible mechanisms underlying the antibacterial activity of Ag NPs, the changes in energy-dependent H^+^-fluxes through the bacterial membrane of *E. coli* (both ATCC 25922 and kanamycin-resistant *E. coli* pARG-25 strains) in the presence of Ag NPs and *R. nigrum* leaf extract were investigated.

### 4.13. Determination of F_O_F_1_-ATPase Activity in Membrane Vesicles in the Presence of Ag NPs and R. nigrum Leaf Extract

The impact of Ag NPs at the H^+^-translocating F_O_F_1_-ATPase activity was investigated in *E. coli* ATCC 25922 and *E. coli* pARG-25 strains to identify possible targets. ATPase activity assay was performed in bacterial membrane vesicles, which were obtained by the Kaback method [48,56]. Bacterial cultures were grown in the presence of *R. nigrum* leaf extract and Ag NPs (10 µg/mL). F_O_F_1_-ATPase activity was evaluated by quantifying the amount of inorganic phosphate liberated after adding ATP to membrane vesicles of bacteria. Inorganic phosphate was detected by Tausski and Shorr method. The membrane vesicles of bacteria were incubated with 0.2 mmol/L DCCD for 10 min [48,56]. F_O_F_1_-ATPase activity is expressed in nmol Pi µg^−1^ protein min^−1^. 

## 5. Conclusions

The leaves of *R. nigrum* are widely used in European traditional medicine due to their high content of biologically active compounds. The metabolomic characterization of phenolic components of *R. nigrum* leaf extract affirms that it can serve as an excellent reducing agent due to the presence of large amounts of flavan-3-ols, hydroxycinnamates, flavonols, quercetin and quercetin derivatives. 

Ag NPs synthesized with the use of *R. nigrum* extract have exhibited excellent antibacterial activity possibly due to the relatively small size and round shapes of nanoparticles. The identified substances can also serve as stabilizers for the synthesized nanoparticles and enhance their shelf life. We suggest that these biogenic Ag NPs can exhibit enhanced antibacterial properties through changes in membrane permeability as a result of the impact of these particles on the H^+^-translocating ATPase activity, energy-dependent H^+^-fluxes and formate hydrogenlyase (FHL) proton-potassium transporter. This phenomenon not only explains the antibacterial potential of *R. nigrum* extract-mediated Ag NPs but also suggests their potential application in biomedicine.

## Figures and Tables

**Table 1:** Phytochemicals tentatively identified by LC-Q-Orbitrap-HRMS in *R. nigrum* extract.

No.	RT (min)	Tentative Identification	Molecular Formula	Molecular Weight	Λmax (nm)	Theoretical (*m/z*)	Observed (*m/z*)	Mass Error (ppm)	Fragments (*m/z*)
1.	5.96	B-typeprocyanidin dimer	C_30_H_26_O_12_	578.14243	279	577.13460	577.13547	−1.49	125.02; 289.07; 407.08
2.	6.23	(epi)Gallocatechin	C_15_H_14_O_7_	306.07396	272	305.06613	305.06659	−1.49	125.02; 137.02; 167.03
3.	6.92	B-typeprocyanidin dimer	C_30_H_26_O_12_	578.14243	281	577.13460	577.13533	−1.25	125.02; 289.07; 407.08
4.	7.28	Caffeoylquinic acid	C_16_H_18_O_9_	354.09509	325	353.08726	353.08772	−1.29	191.05
5.	7.54	(+)-Catechin	C_15_H_14_O_6_	290.07904	279	289.07122	289.07171	−1.69	109.03; 123.04; 125.02; 151.04
6.	8.40	B-typeprocyanidin dimer	C_30_H_26_O_12_	578.14243	279	577.13461	577.13531	−1.21	125.02; 289.07; 407.08
7.	9.30	(-)-Epicatechin	C_15_H_14_O_6_	290.07904	279	289.07122	289.07163	−1.41	109.03; 123.04; 125.02; 151.04
8.	9.93	A-typeprocyanidin trimer	C_45_H_36_O_18_	864.19017	279	863.18235	863.18375	−1.62	289.07; 451.10; 573.10; 711.13
9.	10.42	Rhamnetin glucoside	C_22_H_22_O_12_	478.11113	366	477.10331	477.10395	−1.34	315.05
10.	10.55	A-typeprocyanidin tetramer	C_60_H_50_O_24_	1154.26921	279	1153.26139	1153.26251	−0.97	575.12; 865.20; 1001.21
11.	11.53	Lariciresinol glucoside	C_26_H_34_O_11_	522.21012	280	521.20229	521.20294	−1.24	359.15
12.	12.48	Quercetin rutinoside	C_27_H_30_O_16_	610.15339	354	609.14557	609.14633	−1.25	301.03
13.	12.95	Coumaric acid derivative	C_25_H_28_O_13_	536.15299	311	535.14517	535.14578	−1.13	147.04; 163.04
14.	13.25	Quercetin hexoside	C_21_H_20_O_12_	464.09548	354	463.08766	463.08829	−1.38	300.03; 301.03
15.	13.40	Quercetin glucuronide	C_21_H_18_O_13_	478.07474	354	477.06692	477.06729	−0.76	151.00; 178.99; 301.03
16.	13.50	Epicatechin gallate	C_22_H_18_O_10_	442.09000	279	441.08218	441.08274	−1.27	169.01; 289.07
17.	14.36	Quercetin pentoside	C_20_H_18_O_11_	434.08492	354	433.07709	433.07765	−1.28	300.03; 301.03
18.	14.49	Quercetin malonyl glucoside	C_24_H_22_O_15_	550.09588	354	549.08805	549.08885	−1.45	301.03
19.	14.77	Quercetin pentoside	C_20_H_18_O_11_	434.08492	351	433.07709	433.07752	−0.98	300.03; 301.03
20.	15.09	Quercetin pentoside	C_20_H_18_O_11_	434.08492	347	433.07709	433.07755	−1.05	300.03; 301.03
21.	15.47	Quercitrin	C_21_H_20_O_11_	448.10057	349	447.09274	447.09332	−1.29	300.03; 301.03
22.	15.59	B-type galloylated procyanidin dimer	C_37_H_30_O_16_	730.15339	279	729.14557	729.14679	−1.69	289.07; 407.08
23.	16.60	Coumaric acid derivative	C_20_H_28_O_9_	412.17334	334	411.16551	411.16622	−1.71	145.03; 163.04
24.	17.15	Coumaric acid derivative	C_20_H_28_O_9_	412.17334	310	411.16551	411.16616	−1.57	119.05; 145.03; 163.04
25.	17.33	B-type digalloylated procyanidin dimer	C_44_H_34_O_20_	882.16435	278	881.15653	881.15809	−1.78	287.06; 407.08;729.15
26.	19.54	Musizin glucoside	C_19_H_22_O_8_	378.13147	334	377.12365	377.12408	−1.17	215.07
27.	20.73	Marmesin glucoside	C_20_H_24_O_9_	408.14204	336	407.13421	407.13468	−1.17	230.06; 245.08
29.	22.42	Musizin acetyl glucoside	C_21_H_24_O_9_	420.14204	334	419.13421	419.13471	−1.187	215.07
30.	23.30	Marmesin acetyl glucoside	C_22_H_26_O_10_	450.1526	337	449.14478	449.14526	−1.097	245.08

## Data Availability

The datasets and materials used and/or analyzed during the current study are available from the author upon reasonable request.
